# Accurate Model-Based Point of Gaze Estimation on Mobile Devices

**DOI:** 10.3390/vision2030035

**Published:** 2018-08-24

**Authors:** Braiden Brousseau, Jonathan Rose, Moshe Eizenman

**Affiliations:** 1 Department of Electrical and Computer Engineering, University of Toronto, Toronto, ON M5S 3G4, Canada; 2 Ophthalmology and Vision Sciences, University of Toronto, Toronto, ON M5T 3A9, Canada; 3 Institute of Biomaterials and Biomedical Engineering, University of Toronto, Toronto, ON M5S 3G9, Canada

**Keywords:** Eye Tracking, Gaze Estimation, Mobile Computing, Mobile Eye-Tracking, Gaze-Based Interaction

## Abstract

The most accurate remote Point of Gaze (PoG) estimation methods that allow free head movements use infrared light sources and cameras together with gaze estimation models. Current gaze estimation models were developed for desktop eye-tracking systems and assume that the relative roll between the system and the subjects’ eyes (the ’R-Roll’) is roughly constant during use. This assumption is not true for hand-held mobile-device-based eye-tracking systems. We present an analysis that shows the accuracy of estimating the PoG on screens of hand-held mobile devices depends on the magnitude of the R-Roll angle and the angular offset between the visual and optical axes of the individual viewer. We also describe a new method to determine the PoG which compensates for the effects of R-Roll on the accuracy of the POG. Experimental results on a prototype infrared smartphone show that for an R-Roll angle of $90^{{}^{\circ}}$, the new method achieves accuracy of approximately $1^{{}^{\circ}}$, while a gaze estimation method that assumes that the R-Roll angle remains constant achieves an accuracy of $3.5^{{}^{\circ}}$. The manner in which the experimental PoG estimation errors increase with the increase in the R-Roll angle was consistent with the analysis. The method presented in this paper can improve significantly the performance of eye-tracking systems on hand-held mobile-devices.

## 1. Introduction

Remote eye tracking systems that measure the point of gaze (PoG) have been used in many domains including the measurement of advertising efficacy [1,2], reading studies [3,4,5], and human-machine interfaces [6]. Applications in these domains have been demonstrated largely on specialized, stationary and expensive eye tracking devices. The development of hand-held eye tracking systems date back to the 2000s [7,8] when research groups built fully-custom devices. Recent work has begun to bring eye-tracking technology to widely available, and less expensive mobile devices including smart phones and tablets. This has brought exciting applications that are explicitly designed with mobile context in mind [9,10].

Eye tracking on hand-held devices bring significant challenges beyond those in traditional desktop systems. Key among these is the movement of the eye-tracking device relative to the subject: during the operation of a mobile eye tracker the distance between the device and the user can vary by a factor of 2–3 and the roll angle of the device relative to the user’s eyes (R-Roll) can routinely change by 90${}^{{}^{\circ}}$. Using an eye tracking model robust to these types of movements is essential for high performance in a mobile context. In addition when considering smart phones, the small size of a screen requires more accurate PoG estimates if one wanted to differentiate between the same number of regions as on a larger display such as on a tablet.

Recent mobile eye tracking work has largely focused on unmodified commercial devices [11,12,13,14,15,16,17,18]. The eye-tracking systems on these devices are based on the analysis of eye images from the mobile devices’ RGB cameras where the eyes are not illuminated by specialized light sources (e.g., infrared).

These systems use geometric projections of the boundary between the iris and the limbus, the distance between the iris center and a corneal reflection or machine learning appearance-based methods to calculate the PoG. Two dimensional geometric appearance methods, such as limbus back back-projection [6,19,20] use the eccentricity of the ellipse that defines the boundary between the iris and the sclera to estimate the visual axis of the eye. The more elliptical this boundary becomes, the greater the deviation between the visual axis of the eye and the optical axis of the camera, which can be converted into an estimate of the PoG. This technique is simple to implement, but it is least accurate when there are small deviations between the optical axis of the eye and the camera, which is nearly always the case on the small screen of a smartphone. In a study with a gaze estimation system that used an 11-inch tablet (with a screen-size of 27 × 18 cm) the reported gaze estimation error was 6${}^{{}^{\circ}}$ when the user’s eye was at a distance of 20 cm from the camera [11]. A similar configuration was used in [21] but only achieved 15${}^{{}^{\circ}}$ accuracy. A method that uses the distance between the iris center and a corneal reflection to calculate the PoG is described in [13]. In this method a glint (virtual image created by the front surface of the eye’s cornea) is created by the information displayed on the screen of the mobile device. This method can achieve a point of gaze with an accuracy of 2.9${}^{{}^{\circ}}$. While these approaches can work on an unmodified device they are not robust to any device motion after calibration, especially device roll. If a subject were to rotate the device or reposition their head relative to the device the gaze estimation accuracy would significantly worsen until calibration was redone.

Machine learning appearance-based approaches to mobile gaze estimation have some robustness to natural head and devices movements (insofar as they are represented in the training data). An approach using random forests on an unmodified tablet [14] has demonstrated 3.1 cm gaze estimation error (which is 5.9${}^{{}^{\circ}}$ at 30 cm distance). Using deeper neural networks such as those found in [6,12] a gaze estimation error as low as 1.34 cm has been reported on a standard iPhone. If one assumes an average distance of 20 cm between the eye tracker and the user’s eye (which we base on an analysis of the images in the data set and known field of view ranges for modern iPhone cameras) this error is equivalent to about 3.8${}^{{}^{\circ}}$.

The key advantages of mobile eye-tracking systems that use visible light techniques is that they don’t require special hardware. However, these systems have not yet been able to achieve the accuracy of desktop infrared model-based gaze estimation systems which report 0.5${}^{{}^{\circ}}$ estimation accuracy [22,23,24]).

Artificial infrared illumination in eye tracking has been widely adopted in indoor settings by commercial companies such and has been shown to produce high quality eye tracking systems with under 1${}^{{}^{\circ}}$ of estimation error in a variety of conditions [22,23,24]. Using artificial illumination, the eye model-based approach [22] provides the best performance when the head is free to move relative to the eye-tracker. To achieve this performance, gaze estimation requires an infrared camera and infrared LEDs not currently available on standard mobile devices. However, recently, Apple Inc. (Cupertino, CA, USA) has integrated an infrared camera and infrared light sources in a commercial mobile device (iPhone X) to enhance face tracking and identification algorithms. With only slight modifications, this approach could also support eye tracking methods that use infrared gaze estimation models to calculate the PoG. Many other smart-phone manufacturers are experimenting with native infrared cameras. Google’s Tango development smartphone [25] has a usable infrared camera (although on a back-facing camera), some modern Samsung devices have front facing sensors for iris scanning which use an infrared camera and the prototype device we use in this work, which was provided by Huawei Inc. (Shenzhen, China), has a front-facing infrared camera. We believe infrared front facing cameras may become a standard feature of devices in the near future and one use for them could be artificially illuminated gaze estimation in conjunction with other gaze estimation techniques. We hope that our work helps motivate these changes.

Therefore, in contrast to previous works, our approach, is to assume that infrared illumination and infrared cameras will soon be integrated into commercial mobile devices and by using a model-based method to estimate the PoG achieve accuracy that is similar to that of desktop methods [22,23,24].

This paper presents a novel model-based method for the estimation of the PoG on displays of mobile devices. Since a mobile device can be moved freely in the hands of subjects, several issues arise in PoG estimation on mobile devices that are not as present in stationary desktop systems. All subjects gave their informed consent for inclusion before they participated in the study. The first issue is associated with the need to use more than one coordinate system to describe all the parameters of the gaze estimation model. In desktop systems all model parameters (camera and light sources locations, eye-parameters, display parameters, etc.) are measured in one fixed world coordinate system. In an eye-tracking system that is free to move, some model parameters are continuously changing with the movements of the device (e.g., camera and light source locations) and some model parameters are measured only once in a fixed coordinate system (such as the subjects’ eye parameters). Therefore, for PoG estimation on mobile devices, model parameters that are measured in different coordinate systems must be transformed into a single coordinate system for the estimation of the PoG.

The second issue that arises relates to the common assumption that the relative roll angle between the eye tracking system (which is comprised of the camera, the light sources and the display) and the subjects’ eyes (R-Roll) is approximately 0${}^{{}^{\circ}}$ [22]. This is a reasonable assumption for stationary desktop eye-tracking systems where the head is approximately erect, but not for hand-held eye tracking systems.

We focus in this work on extending an infrared model-based approach found in [22] however the theory provided here is applicable to any hand-held system that may experience R-Roll and computes the PoG using a reconstruction of the optical and visual axis of the eye, even if that is done in the visible light domain.

The objectives of this paper are to (a) describe a novel method that will enable the use of accurate model-based estimation of the PoG on mobile devices; (b) extend the model of [22] by incorporating the R-Roll in the computation of the PoG and (c) provide experimental data to demonstrate the importance of such an extension in mobile devices with limited screen-sizes. The paper is organized as follows: Section 2.1 provides a description of a model-based method for the estimation of the PoG on mobile devices. Section 2.2 presents an analysis of the expected changes in the estimation of the PoG as a function of the R-Roll between the eye tracking system and the subjects’ eyes. Section 2.3 describes the experimental methodology used and Section 3 shows results of these experiments with a prototype industrial device. Finally, in Section 4 we discuss the results and additional sources of errors that affect the estimation of the PoG on mobile devices.

## 2. Materials and Methods

### 2.1. Mathematical Model

**Figure 1:**
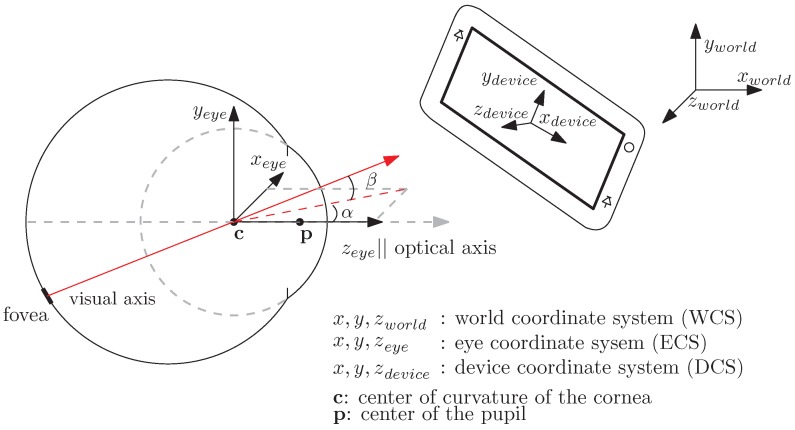
Mobile Eye Tacking System.

This work is based on the model for PoG estimation developed in [22] for desktop systems, which we will refer as the *prior* model. The prior model estimates the PoG on a desktop stationary display by determining the intersection of the visual axis of the eye with the display. To determine unit vectors in the direction of the visual axis of the eye this model first estimates the direction of the optical axis of the eye and then uses the fact that the angular separation between the optical and visual axes is constant to calculate the direction of the visual axis. The direction of the optical axis of the eye (Figure 1) is defined by a line connecting the center of curvature of the cornea, c, and the center of the pupil, p. As shown in the prior model, the direction of the optical axis, ω, is given by:

(1) $$\omega \equiv \frac{p-c}{∥p-c∥}=\left[\begin{array}{c} sin\left(\theta_{eye}\right)cos\left(\phi_{eye}\right)\\ sin(\phi_{eye})\\ -cos\left(\theta_{eye}\right)cos\left(\phi_{eye}\right) \end{array}\right]$$

where p and c are measured in a right handed 3-D Cartesian World Coordinate System (WCS) and $\theta_{eye}$ and $\phi_{eye}$ are the horizontal (yaw) and vertical (pitch) angles of the eye (not shown in Figure 1) with respect to this system. The pupil center, p, and the center of curvature of the cornea, c, are estimated by the prior model using the coordinates of the pupil-center and the virtual images of the light sources that illuminate the eye (corneal reflections) in images from the camera. The visual axis of the eye, ν, also illustrated in Figure 1, is defined by a line connecting the center of curvature of the cornea, c, and the fovea, the highest acuity region of the retina. In the eye coordinate system, the visual axis has a fixed horizontal and vertical angular offsets ($\alpha_{eye}$, $\beta_{eye}$) from the optical axis. These offsets are subject-specific and are estimated, in the prior model, with respect to the horizontal and vertical axes of the WCS during subject calibration procedures. The direction of the visual axis, ν, is given by:

(2) $$\nu =\left[\begin{array}{c} sin\left(\theta_{eye}+\alpha_{eye}\right)cos\left(\phi_{eye}+\beta_{eye}\right)\\ sin(\phi_{eye}+\beta_{eye})\\ -cos\left(\theta_{eye}+\alpha_{eye}\right)cos\left(\phi_{eye}+\beta_{eye}\right) \end{array}\right]$$

In the prior model, all parameters are defined in a fixed world coordinate system (WCS) that is rigidly attached to the display of the system where the *x*-axis and *y*-axis of the WCS are parallel to the rows and the columns of the display and the *z*-axis is perpendicular to the display and pointing towards the subject.

In a mobile eye tracking system, if the pupil center, p, and the center of curvature of the cornea, c, are estimated in a device coordinate system (DCS) that is rigidly attached to the mobile device, Equation (1) can be used to describe the direction of the optical axis of the eye in this coordinate system. In such a system, p and c can be estimated with the prior model using estimates of the pupil-center and corneal reflection locations in images from the mobile device camera. Similar to the definition of the WCS in [22], the mobile device coordinate system is defined as a right-handed 3-D Cartesian coordinates system whose $X_{DCS}$, and $Y_{DCS}$ axes are parallel to the rows and columns of the display of the mobile device, the XY-plane is coincident with the plane of the display and the Z-axis is perpendicular to the display pointing from the display towards the subject. To determine the PoG on the screen (i.e., the intersection of the visual axis of the eye with the screen), a unit vector in the direction of the visual axis, ν, must be calculated in the DCS. Note, however, that since the DCS is continuously moving, $\alpha_{eye}$ and $\beta_{eye}$ (subject-specific eye parameters that were estimated, in the prior model, with respect to the horizontal and vertical axes of the WCS) are not necessarily measured in the DCS so one cannot use Equation (2) to estimate the direction of the visual axis.

To help in the estimation of, $\nu^{DCS}$, (the direction of the visual axis in the DCS) we define an Eye Coordinate System (ECS) that is rigidly attached to the eye (see Figure 1). In such a system the angle between the optical and visual axes does not change with eye rotations or translations. If the ECS is defined as a right handed 3-D Cartesian coordinate system whose axes are labeled $x_{eye}$, $y_{eye}$, $z_{eye}$ where the $z_{eye}$-axis is coincident with the optical axis of the eye, pointing forwards out of the eye and towards the world, $\nu^{ECS}$ can be written as:

(3) $$\nu^{ECS}=\left[\begin{array}{c} sin\left(\alpha_{eye}\right)cos\left(\beta_{eye}\right)\\ sin(\beta_{eye})\\ cos\left(\alpha_{eye}\right)cos\left(\beta_{eye}\right) \end{array}\right]$$

where $\alpha_{eye}$ and $\beta_{eye}$ are the horizontal and vertical angular offsets between the optical and visual axes. Using Equation (3) we can formally write:

(4) $$\nu \equiv \nu^{DCS}=R_{ECS}^{DCS}*\nu^{ECS}$$

where $R_{ECS}^{DCS}$ is the rotation matrix of the ECS with respect to the DCS and can be expressed as

(5) $$R_{ECS}^{DCS}=\left[\begin{array}{ccc} x_{eye}^{DCS} & y_{eye}^{DCS} & z_{eye}^{DCS} \end{array}\right].$$

The notation $x_{eye}^{DCS}$ corresponds to a unit vector in the direction of the $x_{eye}$-axis but described in the device coordinate system. Note that by definition the unit vector in the direction of $z_{eye}$-axis of the ECS with respect to the DCS is given by

(6) $$z_{eye}^{DCS}=\omega ,$$

**Figure 2:**
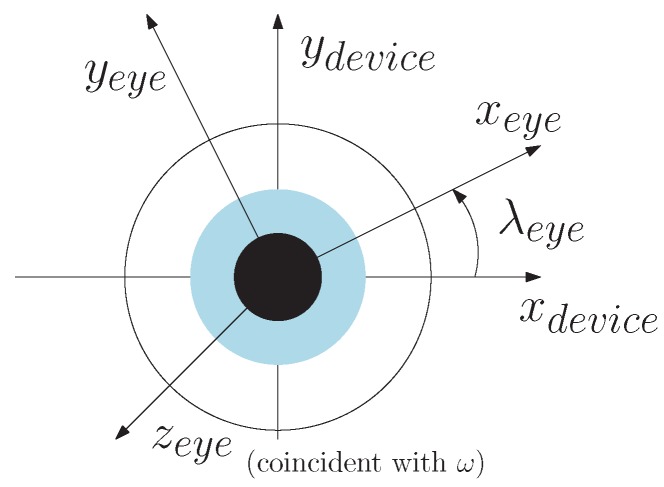
Illustration of $\lambda_{eye}$ translation from DCS to ECS.

If the R-Roll (the rotation angle of the $x_{eye}$-axis and $y_{eye}$-axis with respect to the DCS, $\lambda_{eye}$, in Figure 2) is assumed to be $0^{{}^{\circ}}$ (this assumption will be relaxed later on in the derivation) then a unit vector in the direction of the $x_{eye}^{DCS}$-axis of the ECS with respect to the DCS is:

(7) $$x_{eye,\lambda =0}^{DCS}=\frac{y_{device}^{DCS}\times \omega }{∥y_{device}^{DCS}\times \omega ∥}$$

where

(8) $$y_{device}^{DCS}=\left[\begin{array}{c} 0\\ 1\\ 0 \end{array}\right]$$

is the unit vector in the direction of the Y-axis of the DCS. Next, a unit vector in the direction of the $y_{eye}$-axis of the ECS with respect to the DCS for $\lambda_{eye}=0$ is:

(9) $$y_{eye,\lambda =0}^{DCS}=z_{eye}^{DCS}\times x_{eye,\lambda =0}^{DCS}.$$

When the R-Roll angle between the DCS and the ECS is $\lambda_{eye}$ (i.e., we no longer assume that the R-Roll angle is $0^{{}^{\circ}}$), the direction of the unit vectors in the rotation matrix of the ECS with respect to the DCS can be calculated by:

(10) $$x_{eye}^{DCS}=cos\left(\lambda_{eye}\right)x_{eye,\lambda =0}^{DCS}+sin\left(\lambda_{eye}\right)y_{eye,\lambda =0}^{DCS}$$

and,

(11) $$y_{eye}^{DCS}=-sin\left(\lambda_{eye}\right)x_{eye,\lambda =0}^{DCS}+cos\left(\lambda_{eye}\right)y_{eye,\lambda =0}^{DCS}$$

In summary, the method to estimate the PoG on displays of mobile devices first estimates the direction of the optical axis of the eye in the device coordinate system using (Equation (1)). Then, using values for $\alpha_{eye}$, $\beta_{eye}$, $\lambda_{eye}$ and Equations (3)–(11) an estimate of the direction of the visual axis in the DCS is determined. Finally, the intersection of a vector aligned with the visual axis and the display, $Z_{dcs}=0$ is calculated, providing the PoG estimate on the display of the mobile device.

### 2.2. Quantifying the Effects of Relative Roll on POG Estimation

In this section we derive an expression for the difference between the estimated PoGs when $\lambda_{eye}=0^{{}^{\circ}}$ (using Equations (1) and (3)–(11)) and when $\lambda_{eye}=$ R-Roll (using Equations (1) and (3)–(11)). The purpose of this derivation is to determine the model parameters that mediate the effects of the R-Roll angle on the estimation of the PoG and to determine the expected magnitude of these effects. To simplify the derivation, the pitch and yaw angles of the eye were set to $0^{{}^{\circ}}$.

Let $\nu^{0}$ be the direction of the visual axis when $\lambda_{eye}$ is set to 0 and ν the direction of visual axis when $\lambda_{eye}=$ R-Roll. From Section 2.1 we have

(12) $$\nu^{0}=R_{ECS}^{DCS}\left(\lambda_{eye}=0^{{}^{\circ}}\right)\nu^{ECS}$$

and,

(13) $$\nu =R_{ECS}^{DCS}\left(\lambda_{eye}\right)\nu^{ECS}$$

where

(14) $$R_{ECS}^{DCS}\left(\lambda_{eye}=0^{{}^{\circ}}\right)=\left[\begin{array}{ccc} 1 & 0 & 0\\ 0 & 1 & 0\\ 0 & 0 & -1 \end{array}\right]$$

and,

(15) $$R_{ECS}^{DCS}\left(\lambda_{eye}\right)=\left[\begin{array}{ccc} cos(\lambda_{eye}) & -sin(\lambda_{eye}) & 0\\ sin(\lambda_{eye}) & cos(\lambda_{eye}) & 0\\ 0 & 0 & -1 \end{array}\right]$$

The angular difference between ν and $\nu^{0}$, δ, can be obtained from:

(16) $$\nu \cdot \nu^{0}=\left|\nu \right|\left|\nu^{0}\right|cos\left(\delta \right).$$

Substituting Equations (12) and (13), into (16) and using Equation (3) (noting that both ν and $\nu_{0}$ are unit vectors), yields δ as a function of $\lambda_{eye}$, $\alpha_{eye}$, $\beta_{eye}$.

(17) $$\begin{array}{cc} \delta =cos^{-1}[ & cos\left(\lambda_{eye}\right)\left[sin^{2}\left(\alpha_{eye}\right)cos^{2}\left(\beta_{eye}\right)+sin^{2}\left(\beta_{eye}\right)\right]\\ & +cos^{2}\left(\alpha_{eye}\right)cos^{2}\left(\beta_{eye}\right)]. \end{array}$$

**Figure 3:**
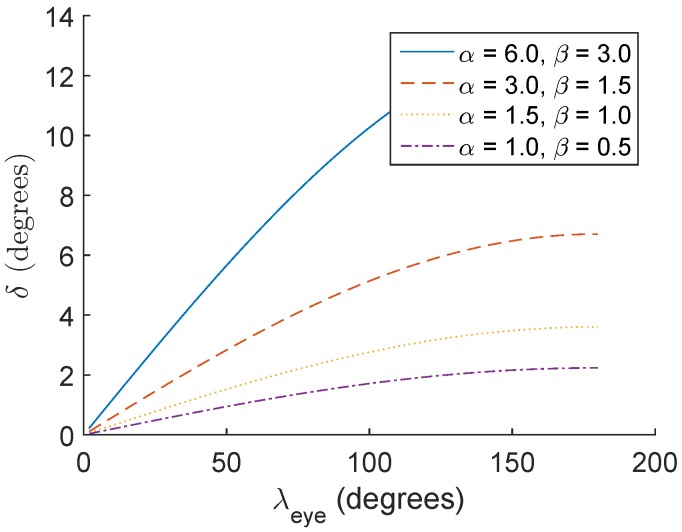
Expected estimation error (δ) as a function of the R-Roll angle ($\lambda_{eye}$) for four different angular offsets (α and β) between the optical and visual axes.

Figure 3 shows the expected difference between the directions of the visual axes, $\delta^{{}^{\circ}}$, when $\lambda_{eye}$ is set to $0^{{}^{\circ}}$ (the assumption of the prior model) and when $\lambda_{eye}$ is set to R-Roll. In Figure 3, $\lambda_{eye}$ changes from 0 to 180 degrees. The four curves in Figure 3 are for four values of $\alpha_{eye}$ and $\beta_{eye}$. These values were selected to span the full range of expected angular offsets between the optical and visual axes in adults. The curve for $\alpha_{eye}=3.0^{{}^{\circ}}$ and $\beta eye=1.5^{{}^{\circ}}$ shows the expected effects of the R-Roll angle on the estimated direction of the visual axis for an average adult human eye. For an average eye, during an orientation change of the mobile device from landscape to portrait ($\lambda_{eye}=90^{{}^{\circ}}$) the value of δ is $4.7^{{}^{\circ}}$.

If the eye is at a distance of 30 cm from the display this orientation change would result in a PoG estimation error of approximately 2.5 cm, or more than one third the display-width of a typical smart phone. Figure 3 also shows that individuals with larger offsets between the optical and visual axes are expected to have larger differences between the directions of their estimated visual axis (δ) due to R-Roll. Moreover, the change in δ is approximately linear for a large range of R-Roll angle values, $\lambda_{eye}$. The relatively modest slopes of the lines in Figure 3 (1/50 to 1/11), indicate that the sensitivity of δ to errors in the estimation of $\lambda_{eye}$ is small. For an average eye, a $1^{{}^{\circ}}$ error in the estimation of $\lambda_{eye}$ will affect the estimation of the direction of the visual axis by only $0.04^{{}^{\circ}}$.

### 2.3. Experimental Procedure

**Figure 4:**
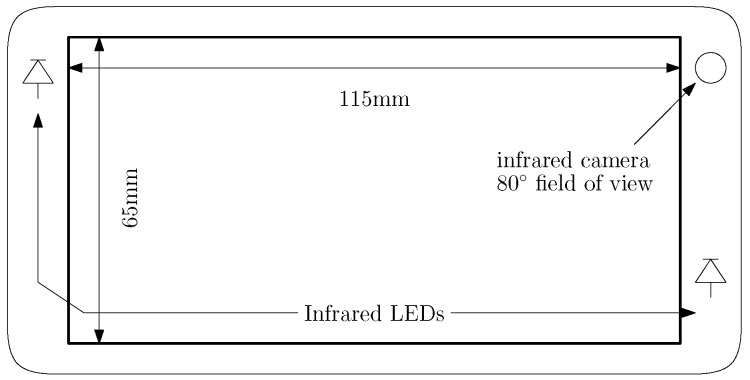
Prototype IR mobile device.

**Figure 5:**
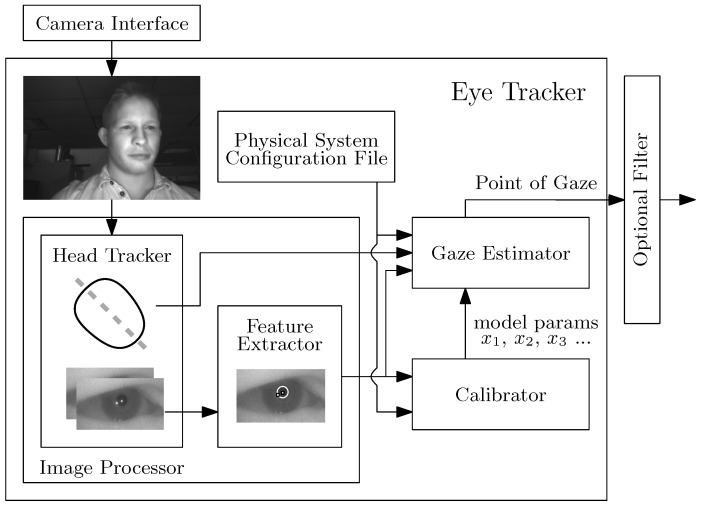
Eye Tracking Modules and Software Flow.

We conducted a study with four subjects to determine the performance of the model-based gaze estimation method in the presence of R-Roll that was developed in Section 2.1. The four subjects looked at targets on the display of a mobile device while the R-Roll angle was configured to one of 3 angles, 0, 45, and 90 degrees. The mobile device that we used in the experiments is a prototype that was provided by Huawei Corporation. It employs an Android-based operating system and has two infrared LEDs, a 4K front-facing infrared camera with a 80${}^{{}^{\circ}}$ field of view and a 5 inch display as shown in Figure 4. An 80${}^{{}^{\circ}}$ field of view is larger than average for the front facing camera of a typical smartphone and will increase the likelihood that the subject’s eye are in the frame. The full software eye-tracking system, illustrated in Figure 5, has the following components: (a) a head and facial feature tracker that was used to determine the location of the left and right eye regions in images of the infrared camera; (b) an image processing system to estimate the locations of the pupil center and corneal reflections (of the infrared LEDs) in each eye region; and (c) a gaze estimation model. The gaze estimation model uses parameters that describe the location and size of relevant components on the prototype device, the user’s eye (e.g., $\alpha_{eye}$ and $\beta_{eye}$) and the R-Roll angle ($\lambda_{eye}$).

**Figure 6:**
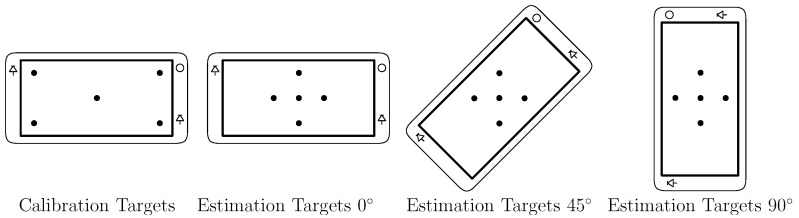
Calibration and Estimation Targets.

During the experiment, four videos from the camera of the eye-tracking system were collected for each of the four subjects in the following way: the device was held in a stand capable of being set at a fixed roll angle, and the head of the subject was positioned on a chin rest at a distance of 30 cm from the device. The chin rest and device stand acts to minimize several sources of variance on the final gaze estimation error that could result from head or device movements. Using a chin rest in this way allows us to isolate the change in gaze estimation error due only to R-Roll between the subject and the device. During the recording of each video, subjects looked at five known targets on the display as shown in Figure 6. The first video (during which calibration is performed) was used to determine subject-specific eye parameters that included $\alpha_{eye}$ and $\beta_{eye}$. These parameters, together with parameters that describe the optical and physical characteristics of the system (the location of the LEDs and camera) and estimates of the physical locations of the pupil center and the corneal reflections were used to generate estimates of the direction of the optical axis. These estimates were generated by the gaze estimation model described in [23]. Then, using the estimated direction of the optical axis, the measured head tilt (measured by the head-tracker) and the method described in Section 2.1 the PoGs in three subsequent videos were computed. These videos were recorded with three set R-Roll angles: 0, 45 and 90 degrees relative to the orientation of the eye-tracking system during the calibration video. This rotation was archived by rotating the mobile device with respect to gravity while the subject head orientation remained fixed with a chin rest. At each R-Roll angle, subjects were instructed to gaze at five fixation targets for 5 s (150 frames) each.

In general, the R-Roll angle is a combination of the head tilt angle with respect to the device and the eye’s counter-roll [26]. In the experiments described in this here the R-Roll angle between the eye and the eye-tracker is determined by the head tilt since the eyes’ counter-roll eye movements were minimized by supporting the subject’s head on a chin rest. Based on our theoretical predictions from (17) we don’t expect counter roll to be a significant source of error however for subjects sitting upright. In the experiments head tilt was measured by the system’s head tracker; we used one provided by Visage Technologies.

PoG estimates for each frame were computed (If the pupil center and corneal reflection of the eyes were detected by the feature extractor in that frame) using one of the two following methods: Method 1 assumed that the R-Roll angle was $0^{{}^{\circ}}$ when estimating the PoG (i.e., $\lambda_{eye}$ was set to $0^{{}^{\circ}}$, for all test conditions) and Method 2 where $\lambda_{eye}$ was set to the R-Roll that was measured by the head tracker. Comparing the results of the two methods provides a direct estimate of the improvement to the accuracy of PoG estimation when the R-Roll angle is used by the model. Since both methods used the same estimates of pupil center and corneal reflections (because these results are computed through off-line processing of the recorded videos), we can ensure that the only difference between the PoG estimates of the two methods is associated with the use of the R-Roll angle in the computation.

The PoG estimation error for each sample video was computed by first calculating the average absolute distance between the PoG estimates and the position of the fixation target for these estimates and averaging the errors of the 5 targets.

## 3. Experiment Results

**Figure 7:**
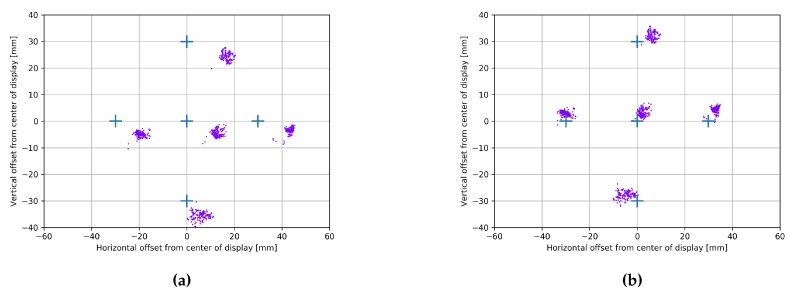
Gaze estimates for subject 02 at 30 cm device distance and $\lambda_{eye}=90^{{}^{\circ}}$. The 5 crosses represent the fixation points. The point [0,0] is the center of the display. (**a**) PoG estimates computed with Method 1 (no-compensation for R-Roll = 90${}^{{}^{\circ}}$); (**b**) PoG estimates computed with Method 2 (with compensation for the R-Roll = 90${}^{{}^{\circ}}$).

Figure 7a,b show the PoG estimates of one subject (subject 02) when the R-Roll angle is $90^{{}^{\circ}}$. Figure 7a shows the PoG estimates using Method 1 ($\lambda_{eye}$ set to $0^{{}^{\circ}}$) and Figure 7b shows estimates using Method 2 ($\lambda_{eye}$ is estimated by the head-tilt). The crosses in each figure shows the location of the targets, and the scatter plot of dots are the of estimated PoGs. When Figure 7a,b are compared it is clear that when the estimated R-Roll angle is used in the calculations of the PoG the accuracy of the PoG estimation improves.

Table 1 presents the PoG estimation errors for each of the four subjects at three R-Roll angles (0${}^{{}^{\circ}}$, 45${}^{{}^{\circ}}$ and 90${}^{{}^{\circ}}$). The column labeled Method indicates if Method 1 or Method 2 were used in the estimation of the PoG. The results show that the average error for Method 2 is approximately 1 degree and that the magnitude of the errors for the three roll angles are similar. The average error increased by 3.2% (0.92${}^{{}^{\circ}}$ to 0.95${}^{{}^{\circ}}$) when the R-Roll angle chnaged from was 0${}^{{}^{\circ}}$ to 45${}^{{}^{\circ}}$ and by 26% (0.92${}^{{}^{\circ}}$ to 1.16${}^{{}^{\circ}}$) when the R-Roll angle changed from 0${}^{{}^{\circ}}$ to 90${}^{{}^{\circ}}$. When Method 1, in which the R-Roll angle was assumed to remain constant during the experiment (i.e., $\lambda_{eye}$ is set to 0${}^{{}^{\circ}}$), was used for the computation of the PoG, the average error increased by 251% (0.90${}^{{}^{\circ}}$ to 2.26${}^{{}^{\circ}}$) when the R-Roll angle was changed from 0${}^{{}^{\circ}}$ to 45${}^{{}^{\circ}}$ and by 389% (0.90${}^{{}^{\circ}}$ to 3.50${}^{{}^{\circ}}$) when the R-Roll angle was changed from 0${}^{{}^{\circ}}$ to 90${}^{{}^{\circ}}$.

To highlight the importance of using the R-Roll angle in the estimation of the PoG in mobile-device-based eye tracking systems (i.e., limited screen sizes) consider a metric that describes the radius of the circle that includes 95% of the PoG estimates around a fixation target. When Method 2 is used for a 90${}^{{}^{\circ}}$ relative roll, 95% of the estimates are within a radius of 12 mm from the fixation target. When Method 1 is used for 90${}^{{}^{\circ}}$ relative roll, the 95% enclosing radius is much larger, at 28 mm. If one assumes that for applications that use gaze-selection, reliable operation requires that 95% of the estimates are associated with the intended fixation target, the use of Method 2 will allow 45 distinct fixation targets on a 5 inch display while Method 1 will allow for only 8 distinct fixation targets.

Table 2 shows, for each of the four subjects, differences in PoG estimation when $\lambda_{eye}$ is set to 0${}^{{}^{\circ}}$ in the computation of the PoG and when $\lambda_{eye}$ is set to R-Roll angle. The Table shows the differences computed by our model, $\delta_{theory}$, (derived as δ in Section 2.2, Equation (17)) and the experimentally measured differences, $\delta_{measured}$. The last row of the Table gives the absolute difference between $\delta_{measured}$ and $\delta_{theory}$ for each R-Roll angle. The average error between the measured and predicted differences is 0.28${}^{{}^{\circ}}$ which is approximately 15% of the average measured differences (1.82${}^{{}^{\circ}}$). This data provides further evidence that the model in Section 2.1 can be used to accurately determine the PoG when the R-Roll angle changes. In Table 2, the measured $\alpha_{eye}$ and βeye of each subject are presented in the first two rows. As predicted by Equation (17), the magnitude of the measured differences is correlated with the magnitude of the offset between the optical and visual axes ($\alpha_{eye}$ and βeye), with a correlation factor of 0.89 at a R-Roll angle of 90${}^{{}^{\circ}}$.

## 4. Discussion and Conclusions

Using the method that was described in this paper, when the R-Roll angle is approximately 0${}^{{}^{\circ}}$, (which is a testing condition similar to tests on desktop eye-tracking systems) the RMS PoG estimation error on the display of the mobile system is approximately 1.0${}^{{}^{\circ}}$. This is substantially more than the typical 0.5${}^{{}^{\circ}}$ RMS error in desktop systems [22] that use a similar gaze estimation model to calculate the PoG. The main reasons for the larger errors on mobile devices are associated with the difficulty to accurately estimate the pupil center and the corneal reflections locations in images from the camera of the mobile device and the inability to estimate the subject’s specific eye parameters as accurately as it can be done on desktop systems. In mobile systems the combination of smaller size sensors, inferior optics and reduced IR illumination (due to the need to conserve battery power) reduces the quality of eye images from the cameras of these systems when compared with the quality of images from the cameras of desktop systems. In the four subjects that were tested in this study, difficulties to determine eye-features in subject 04 increased the average PoG estimation error for this subject to about 1.4${}^{{}^{\circ}}$, which is significantly higher than the PoG estimation errors in the three other subjects. The reduced precision of the estimated eye features alongside the smaller physical display size of mobile devices leads to a reduced ability to estimate accurately the subject’s specific eye parameters (e.g., $\alpha_{eye}$ and $\beta_{eye}$), as the angular separation between the calibration targets on the display is limited.

The lower accuracy in the estimation of $\alpha_{eye}$ and $\beta_{eye}$ in mobile eye-tracking systems explains some of the differences between the expected and observed changes in gaze estimation due to R-Roll (shown in Table 2). For subjects 02 and 03, the expected and observed changes in gaze estimations due to R-Roll are relatively small (less than 0.25${}^{{}^{\circ}}$) which indicates that estimated subject-specific eye-parameters were relatively accurate. For subject 04, the estimated subject specific eye-parameters are relatively inaccurate and therefore the expected and observed changes due to R-Roll are much larger (1${}^{{}^{\circ}}$ when R-Roll was 90${}^{{}^{\circ}}$). These results suggest that the calibration procedure for small screen mobile eye trackers are particularly vulnerable and should be improved in future iterations of this work.

Another parameter that affects the accuracy of the estimated PoG in smart-phone-based eye-tracking systems is the accuracy of the measured R-Roll angle. In general, the R-Roll angle is a combination of the head tilt angle with respect to the device and the eye’s counter roll [27]. In our experiments the impact of counter roll on the estimation of the R-Roll angle was minimized with the use of a chin rest that minimizes head movements (i.e., minimize the eye’s counter roll movements) and the R-Roll angle was measured by a head-tracker that provided estimates of the head tilt. Noise in the estimation of the head-tilt by the head-tracker that was used in this study is less than 1${}^{{}^{\circ}}$ and therefore, for a typical eye, the effect on PoG estimation due to errors in the estimation of head tilt are relatively small (0.02${}^{{}^{\circ}}$ to 0.09${}^{{}^{\circ}}$). When the head is not supported on a chin rest so the head-tilt can change during the experiment, the R-Roll angle between the eye-tracker and the eyes will be affected by counter roll (torsional eye movements, note that in this study we minimized torsional eye movements by using a chin-rest). Although the amount of counter roll is a non-linear function of head-tilt and it varies between subjects its gain is less than 0.1 [27]. This means that for a subject who chnages posture from standing or sitting to lying horizontally (a change of 90${}^{{}^{\circ}}$), a counter roll of as much as 9${}^{{}^{\circ}}$ might be observed. If the value of the R-Roll angle for this situation was estimated based on the measurement head-tilt from the head tracker (accurate to within a degree), the overall error in $\lambda_{eye}$ could be as high as 10${}^{{}^{\circ}}$. For the range of possible offsets between the optical and visual axes (Figure 3), a 10${}^{{}^{\circ}}$ error in the estimation of $\lambda_{eye}$ would change the PoG estimation by 0.2${}^{{}^{\circ}}$ to 0.9${}^{{}^{\circ}}$.

To obtain more accurate measurements of the R-Roll angle one can measure the rotation of iris structures in images of the eye-tracker’s camera [28]. This technique can provide accurate estimates of the R-Roll regardless of the root cause for the relative movements, whether it is device rotation, head rotation, eye counter-roll or torsional eye movements that are governed by Listings law [29] (rotations in the plane orthogonal to the visual axis as a function of gaze direction). This method, however, is impractical for current-generation mobile devices because of insufficient resolution to detect iris patterns in images from the front facing cameras. Alternatively, one can use a model that describes the typical counter roll as a function of head tilt to estimate the R-Roll angle. In this approach, the orientation of the mobile device relative to gravity would be estimated using the accelerometer on the device and the orientation of the head relative to the device would be measured by the head tracker. When combined, the tilt of the head relative to gravity can be computed and the expected counter roll can be estimated from the model. $\lambda_{eye}$ will then be computed by subtracting the estimated eye counter-roll from the measured head-tilt.

The infrared model-based eye tracking method presented in this paper is tolerant of both head and devices movements and has PoG estimation accuracy of 1${}^{{}^{\circ}}$. The method is much more accurate than methods that do not use models to estimate the point of gaze (limbus back projections and measurement of the distance between the center of the iris and a corneal reflection) and is less sensitive to relative movements between the eyes and the mobile device. When compared or to deep leaning appearance based systems [12], which are robust to relative movements between the eyes and the mobile device the accuracy of the method presented in this paper is significantly better than that reported in [12] (1${}^{{}^{\circ}}$ vs. 3.8${}^{{}^{\circ}}$). That isn’t to say that our approach isn’t without some significant limitations however, as discussed in the following section.

### 4.1. Limitations

The eye tracking gaze-estimation model in this paper can be used to estimate gaze position on screens of smart phones with accuracy of 1 degree. The performance of the gaze estimation model depends on the accuracy in which the pupil-center and the coordinates of the corneal reflections can be estimated.

The ability to accurately estimate these parameters depends on operational parameters such as rapid changes in eye illumination due to rapid changes in the distance between the smartphone and the subject, interference from sunlight, reflections of the IR light from eye-glasses, upper and lower eyelid interference with the pupil-iris boundary and situations in which the subject’s eyes are no longer within the field of view of the smartphone’s camera [30]. Solving these issues is a non trivial task and can be rather specific to the hardware implementation of the eye-tracker (i.e., it is best solved by companies that design eye-tracking systems), but, the work presented in this paper shows that when these issues are solved properly, one can estimate the gaze position on the screen of a smartphone with an accuracy of 1 degree.

One promising approach to overcome some of the above limitations is to augment the gaze estimation model that is described in this paper (i.e., using IR illumination) with a gaze estimation model that relies on ambient illumination. In a gaze estimation model that relies only on ambient illumination the center of the pupil and the center of rotation of the eye in 3D space are used to reconstruct the direction of the optical axis of the eye [31]. Then, the method that is described in this paper can be used to reconstruct the direction of the visual axis and estimate the PoG on the screen of the mobile device.

### 4.2. Conclusions

In summary, in this paper we presented a novel method to determine the PoG on displays of mobile-device-based eye tracking systems. The method uses measurements of the R-Roll angle between the eye-tracking system and the subject’s eyes to provide more accurate PoG estimates when the mobile device is free to rotate in the hands of the user. Using a prototype smartphone-based eye-tracking system we showed that when the R-Roll angle is used in the calculations of the PoG the average error in the estimation of the PoG is approximately 1${}^{{}^{\circ}}$. When the R-Roll between the eye-tracking system and the subject’s eyes is assumed to remain constant during use, the average PoG estimation error when the hand-held device changes from portrait to landscape mode increased to 3.5${}^{{}^{\circ}}$. These results clearly demonstrate that by using the novel method that is described in this paper one can significantly improve the performance of mobile-device based eye tracking systems.

## Figures and Tables

**Table 1:** Gaze estimation errors for R-Roll angles of 0${}^{{}^{\circ}}$, 45${}^{{}^{\circ}}$ and 90${}^{{}^{\circ}}$. In Method 1 $\lambda_{eye}$ set to 0${}^{{}^{\circ}}$ when estimating the PoG while in Method 2 $\lambda_{eye}$ set to the measured R-Roll from the head tracker.

		Average Gaze Error (mm)			Average Gaze Error (Degrees)		
Subject	Method	R-Roll $=0^{{}^{\circ}}$	R-Roll $=45^{{}^{\circ}}$	R-Roll $=90^{{}^{\circ}}$	R-Roll $=0^{{}^{\circ}}$	R-Roll $=45^{{}^{\circ}}$	R-Roll $=90^{{}^{\circ}}$
01	2	5.07	4.7	5.05	0.96	0.89	0.96
	1	4.91	11.11	15.96	0.93	2.12	2.75
02	2	3.73	4.55	5.18	0.71	0.86	0.98
	1	3.52	9.08	14.39	0.67	1.73	2.74
03	2	3.15	4.77	4.12	0.60	0.91	0.78
	1	3.14	11.91	18.42	0.59	2.27	3.51
04	2	7.23	5.94	10.05	1.38	1.13	1.91
	1	7.31	15.44	24.66	1.39	2.94	4.69
**Average**	2	4.80	4.99	6.10	0.92	0.95	1.16
	1	4.72	11.88	18.36	0.90	2.26	3.50

**Table 2:** Predicted and measured differences between PoG estimations when the R-Roll angle is used in the computation of the PoG (Method 2) and when it is assumed to be 0 (Method 1).

Subject	01		02		03		04	
$|\alpha_{eye}|$	1.73		1.21		1.78		2.67	
$|\beta_{eye}|$	0.5		0.28		0.92		0.25	
$\lambda_{eye}$	$45^{{}^{\circ}}$	$90^{{}^{\circ}}$	$45^{{}^{\circ}}$	$90^{{}^{\circ}}$	$45^{{}^{\circ}}$	$90^{{}^{\circ}}$	$45^{{}^{\circ}}$	$90^{{}^{\circ}}$
$\delta_{theory}$	1.37${}^{{}^{\circ}}$	2.54${}^{{}^{\circ}}$	0.94${}^{{}^{\circ}}$	1.75${}^{{}^{\circ}}$	1.53${}^{{}^{\circ}}$	2.83${}^{{}^{\circ}}$	2.06${}^{{}^{\circ}}$	3.81 ${}^{{}^{\circ}}$
$\delta_{measured}$	1.22${}^{{}^{\circ}}$	2.08${}^{{}^{\circ}}$	0.86${}^{{}^{\circ}}$	1.76${}^{{}^{\circ}}$	1.36${}^{{}^{\circ}}$	2.72${}^{{}^{\circ}}$	1.81${}^{{}^{\circ}}$	2.78${}^{{}^{\circ}}$
$|\delta_{theory}-\delta_{measured}|$	0.15${}^{{}^{\circ}}$	0.46${}^{{}^{\circ}}$	0.08${}^{{}^{\circ}}$	0.01${}^{{}^{\circ}}$	0.17${}^{{}^{\circ}}$	0.11${}^{{}^{\circ}}$	0.25${}^{{}^{\circ}}$	1.03${}^{{}^{\circ}}$

## References

[1] Hervet G., Guérard K., Tremblay S., Chtourou M.S. (2011). Is banner blindness genuine? Eye tracking internet text advertising. Appl. Cognit. Psychol..

[2] Resnick M., Albert W. (2014). The Impact of Advertising Location and User Task on the Emergence of Banner Ad Blindness: An Eye-Tracking Study. Int. J. Hum. Comput. Interact..

[3] Rayner K. (1998). Eye movements in reading and information processing: 20 years of research. Psychol. Bull..

[4] Duggan G.B., Payne S.J. Skim Reading by Satisficing: Evidence from Eye Tracking. Proceedings of the SIGCHI Conference on Human Factors in Computing Systems.

[5] Mazzei A., Eivazi S., Marko Y., Kaplan F., Dillenbourg P. 3D Model-based Gaze Estimation in Natural Reading: A Systematic Error Correction Procedure Based on Annotated Texts. Proceedings of the Symposium on Eye Tracking Research and Applications.

[6] Wang J.G., Sung E., Venkateswarlu R. (2005). Estimating the eye gaze from one eye. Comput. Vis. Image Underst..

[7] Vertegaal R., Dickie C., Sohn C., Flickner M. Designing attentive cell phone using wearable eyecontact sensors. Proceedings of the CHI’02 ACM Extended Abstracts on Human Factors in Computing Systems.

[8] Nagamatsu T., Yamamoto M., Sato H. MobiGaze: Development of a gaze interface for handheld mobile devices. Proceedings of the CHI’10 ACM Extended Abstracts on Human Factors in Computing Systems.

[9] Liu D., Dong B., Gao X., Wang H. (2015). Exploiting eye tracking for smartphone authentication. International Conference on Applied Cryptography and Network Security.

[10] Khamis M., Hasholzner R., Bulling A., Alt F. GTmoPass: Two-factor authentication on public displays using gaze-touch passwords and personal mobile devices. Proceedings of the 6th ACM International Symposium on Pervasive Displays.

[11] Wood E., Bulling A. EyeTab: Model-based Gaze Estimation on Unmodified Tablet Computers. Proceedings of the ETRA ’14 Symposium on Eye Tracking Research and Applications.

[12] Krafka K., Khosla A., Kellnhofer P., Kannan H., Bhandarkar S., Matusik W., Torralba A. Eye Tracking for Everyone. Proceedings of the IEEE Conference on Computer Vision and Pattern Recognition (CVPR).

[13] Huang M.X., Li J., Ngai G., Leong H.V. Screenglint: Practical, in-situ gaze estimation on smartphones. Proceedings of the 2017 CHI Conference on Human Factors in Computing Systems.

[14] Huang Q., Veeraraghavan A., Sabharwal A. (2017). TabletGaze: Dataset and analysis for unconstrained appearance-based gaze estimation in mobile tablets. Mach. Vis. Appl..

[15] Kao C.W., Yang C.W., Fan K.C., Hwang B.J., Huang C.P. An adaptive eye gaze tracker system in the integrated cloud computing and mobile device. Proceedings of the 2011 IEEE International Conference on the Machine Learning and Cybernetics (Icmlc).

[16] Holland C., Garza A., Kurtova E., Cruz J., Komogortsev O. Usability evaluation of eye tracking on an unmodified common tablet. Proceedings of the CHI’13 Extended Abstracts on Human Factors in Computing Systems.

[17] Holland C., Komogortsev O. Eye tracking on unmodified common tablets: Challenges and solutions. Proceedings of the Symposium on Eye Tracking Research and Applications.

[18] Ishimaru S., Kunze K., Utsumi Y., Iwamura M., Kise K. Where are you looking at?-feature-based eye tracking on unmodified tablets. Proceedings of the 2013 IEEE 2nd IAPR Asian Conference on Pattern Recognition (ACPR).

[19] Wang J.G., Sung E. (2001). Gaze determination via images of irises. Image Vis. Comput..

[20] Hansen D.W., Pece A.E. (2005). Eye tracking in the wild. Comput. Vis. Image Underst..

[21] Hohlfeld O., Pomp A., Link J.Á.B., Guse D. On the applicability of computer vision based gaze tracking in mobile scenarios. Proceedings of the 17th International Conference on Human-Computer Interaction with Mobile Devices and Services.

[22] Guestrin E., Eizenman M. (2006). General theory of remote gaze estimation using the pupil center and corneal reflections. IEEE Trans. Biomed. Eng..

[23] Guestrin E.D., Eizenman M. Remote Point-of-gaze Estimation Requiring a Single-point Calibration for Applications with Infants. Proceedings of the 2008 Symposium on Eye Tracking Research.

[24] Model D., Eizenman M. (2010). An Automatic Personal Calibration Procedure for Advanced Gaze Estimation Systems. IEEE Trans. Biomed. Eng..

[25] Keralia D., Vyas K., Deulkar K. (2014). Google project tango, a convenient 3D modeling device. Int. J. Curr. Eng. Technol..

[26] Miller E.F. (1962). Counterrolling of the human eyes produced by head tilt with respect to gravity. Acta Oto-Laryngol..

[27] Maxwell J.S., Schor C.M. (1999). Adaptation of torsional eye alignment in relation to head roll. Vis. Res..

[28] Boles W., Boashash B. (1998). A human identification technique using images of the iris and wavelet transform. IEEE Trans. Signal Process..

[29] Hepp K. (1990). On Listing’s law. Commun. Math. Phys..

[30] Khamis M., Baier A., Henze N., Alt F., Bulling A. Understanding Face and Eye Visibility in Front-Facing Cameras of Smartphones used in the Wild. Proceedings of the 2018 CHI Conference on Human Factors in Computing Systems.

[31] Wang K., Ji Q. Hybrid Model and Appearance Based Eye Tracking with Kinect. Proceedings of the Ninth Biennial ACM Symposium on Eye Tracking Research & Applications.

